# Differential Gene Expression in the Heads of Behaviorally Divergent *Culex pipiens* Mosquitoes

**DOI:** 10.3390/insects12030271

**Published:** 2021-03-23

**Authors:** Anna Noreuil, Megan L. Fritz

**Affiliations:** Department of Entomology, University of Maryland, College Park, MD 20742, USA; anoreuil@umd.edu

**Keywords:** *Culex pipiens*, host preference, RNA sequencing, gene expression, whole heads, sensory genes

## Abstract

**Simple Summary:**

Host preference has profound impacts on the epidemiology of mosquito-borne disease transmission, yet much is still unknown about the molecular basis for these preferences. Here, we examined host preference in the West Nile virus vector, *Culex pipiens*. We examined human and avian landing rates for eight populations: five originating from above- and three from below-ground breeding and overwintering habitats. While above-ground populations tended to be biased toward avian landing and below-ground populations tended toward human landing, a range of behaviors was observed, consistent with other mosquito species. Patterns of differential expression and splice site variation were measured for one avian- and one human-seeking population as a first step toward identifying genes involved in regulation of blood feeding behaviors as well as differences in host preference. We conclude with a discussion of specific differentially expressed genes and their potential to influence host seeking behaviors of *Cx. pipiens* females.

**Abstract:**

Host preferences of *Cx. pipiens*, a bridge vector for West Nile virus to humans, have the potential to drive pathogen transmission dynamics. Yet much remains unknown about the extent of variation in these preferences and their molecular basis. We conducted host choice assays in a laboratory setting to quantify multi-day human and avian landing rates for *Cx. pipiens* females. Assayed populations originated from five above-ground and three below-ground breeding and overwintering habitats. All three below-ground populations were biased toward human landings, with rates of human landing ranging from 69–85%. Of the five above-ground populations, four had avian landing rates of >80%, while one landed on the avian host only 44% of the time. Overall response rates and willingness to alternate landing on the human and avian hosts across multiple days of testing also varied by population. For one human- and one avian-preferring population, we examined patterns of differential expression and splice site variation at genes expressed in female heads. We also compared gene expression and splice site variation within human-seeking females in either gravid or host-seeking physiological states to identify genes that may regulate blood feeding behaviors. Overall, we identified genes with metabolic and regulatory function that were differentially expressed in our comparison of gravid and host-seeking females. Differentially expressed genes in our comparison of avian- and human-seeking females were enriched for those involved in sensory perception. We conclude with a discussion of specific sensory genes and their potential influence on the divergent behaviors of avian- and human-seeking *Cx. pipiens*.

## 1. Introduction

Vertebrate blood meals are required by most mosquito species for reproduction. While this requirement is broadly shared across mosquito taxa, the vertebrate class or species from which females acquire blood is not. Host preference, or preferential feeding on one vertebrate host over others, varies between and even within mosquito species [1]. Preferential feeding behavior has profound impacts on mosquito-borne disease transmission [2]; yet despite its epidemiological importance, large gaps still exist in our knowledge of its underlying molecular and physiological mechanisms.

When seeking a host, mosquitoes must detect and respond to host cues, which vary over space and time in their environment. Such cues include carbon dioxide (CO_2_), visual appearance, local increases in temperature and humidity, and odors emitted from skin and breath [3,4,5,6]. Integration of multiple sensory modalities during host-seeking allows mosquitoes to respond to long-, mid-, and short-range host cues [5,6]. At long range, stimulation with CO_2_ activates host-seeking behavior, allows females to orient toward and locate a host, and heightens responses to host-associated visual stimuli [7,8,9,10,11,12]. At closer range, a CO_2_ stimulus increases sensitivity of the olfactory system to skin odors [13]. Host body heat and humidity guide landing [14], while skin odors are thought to ultimately determine host acceptance [6,15].

Detection of heat, humidity, CO_2_, and odor cues relies upon receptors expressed along the dendrites of neurons, which are housed in porous sensory hairs covering the antennae, maxillary palps, and labellum [16,17]. Thermoreceptor proteins, including TRPA1, which is expressed along the dendrites of neurons in thermosensitive sensory hairs, aid in detection of host body heat and direct movement toward hosts [18]. Chemoreceptors expressed along olfactory receptor neurons (ORNs) include odorant receptors (ORs) co-expressed with an essential co-receptor *orco*, and ionotropic receptors (IRs), both of which detect volatiles in the air. Upon entering pores in the sensory hairs, some host volatiles become bound to odorant-binding proteins (OBPs), which either chaperone the odor molecules to their respective receptors or act to degrade them [19,20]. CO_2_ perception, taste, and contact sensation are mediated by gustatory receptors (GRs), which are also expressed on ORNs and other non-ORNs [16,21]. Binding of host-specific odor molecules and tastants to their chemosensory receptors produces electrophysiological signals, which are sent to and processed in the central nervous system, determining responsiveness to a host [6,15].

The mosquito, *Culex pipiens*, is the primary vector of West Nile virus (WNV) in Eastern North America and exists as two morphologically identical bioforms: *molestus* and true *pipiens* [22]. These forms vary with respect to multiple physiological and behavioral traits, reportedly including blood host preference (Table 1). Form *pipiens* is thought to be predominately avian-seeking, while form *molestus* is thought to be mammalian-, and sometimes human-seeking [23,24,25]. In nature, the two forms can hybridize where populations come into contact with one another [26,27,28,29,30]. *Molestus-pipiens* F1 hybrids display indiscriminate feeding behaviors when tested in a laboratory setting, while progeny of F1s backcrossed to *molestus* or *pipiens* have host responses biased toward that of their non-hybrid parent, indicating a genetic basis for this trait [23].

Here, we acquired eight populations of North American *Cx. pipiens*, collected from previously described above-ground form *pipiens* breeding and over-wintering sites and below-ground form *molestus* breeding sites (Table 1 and Table S1). Hereafter, we refer to these populations with respect to where they were collected rather than bioform nomenclature because it is known that gene flow can occur between the forms in North America [27,30]. We used a multi-day host choice assay to quantify the extent of variation in human and avian host preference among above- and below-ground *Cx. pipiens* and tested for differences between populations. As a first step toward elucidating the molecular basis for host preference in this species, we used RNA sequencing to quantify gene expression differences in the heads of one avian- and one human-seeking population. Population-level differences in gene expression between human- and avian-seeking females may shed light on genes that contribute to observed differences in host preference. 

The *molestus* form of *Cx. pipiens* is facultatively autogenous, or able to produce an egg raft in their first gonotrophic cycle without blood feeding (Table 1). Within days of pupal emergence, females enter a gravid physiological state [43]. After deposition of their first egg raft, however, females must seek a host to acquire blood for subsequent reproductive events. The human-seeking below-ground population used in our RNA sequencing experiment was also facultatively autogenous. For this population, we included heads of both gravid females that do not host-seek [57,58,59] and parous females that do as separate treatments in our RNA sequencing experiment. We then compared gene expression profiles of human-seeking females in different physiological states to determine whether any differentially expressed genes could be involved in the regulation of blood feeding behaviors as females transition from a gravid to parous host-seeking state. 

## 2. Materials and Methods

### 2.1. Mosquito Populations

Eight *Cx. pipiens* populations were collected from above-ground (AG) or below- ground (BG) breeding sites in North America (Table S1). Five were initiated from either diapausing adults collected from AG hibernacula or egg rafts collected from AG breeding sites in three different metropolitan areas: Chicago, IL (n = 3; called AG1–AG3), Laurel, MD (n = 1; AG4), and New York City, NY (n = 1; AG5). The remaining three were derived from eggs, larvae, and adults captured in two collection events at BG breeding sites in Calumet, IL ([37]; BG1 and BG2) and Stinson Beach, CA (BG3). All mosquito populations were reared identically throughout the course of our study according to [23], with exception of the blood feeding regime. AG populations were fed 9 parts Na-heparinated goose blood sweetened with 1 part 50% sucrose solution twice per generation for egg production. All BG populations were facultatively autogenous and did not require blood feeding for egg production in their first gonotrophic cycle. Therefore, they were not offered blood to support colony egg production during our study.

### 2.2. Host Landing Assay

To quantify the extent of variation in host preference across multiple AG and BG populations of *Cx. pipiens*, we used the multi-day landing assay described by Fritz et al. [23]. In brief, an unrestrained two- to three-week-old chicken (*Gallus domesticus*) and the unwashed hand of a 24-year-old white female investigator rested opposite one another on platforms in a circular behavioral arena. A 50 g block of dry ice was placed under each platform, each with a 1 cm diameter hole, allowing for release of CO_2_ into the arena at a mean hourly rate of ca. 258 mL/min. This release rate falls within the acceptable range for host attraction for both human- and avian-seeking mosquito species [60]. Host positions in the arena were alternated between testing days, and the investigator also alternated the hand (i.e., right or left) offered each day. Multiple chicks (N = 62) were used throughout the course of the experiment, but individual mosquitoes were always exposed to the same chick across testing days.

Prior to testing, females were allowed to mate and age in 30 × 30 × 30 cm white Bugdorm-1 cages (MegaView Science Co. Ltd., Taiwan). We confirmed that the behavior of BG1 females at two and three weeks post-emergence (PE) did not differ (see Supplemental Methods), which allowed us to wait one extra week PE for deposition of autogenous egg rafts in BG populations. Thereafter, AG females were always tested at two weeks PE, while BG females were tested at three weeks PE. All mosquitoes were offered a 10% sucrose solution and an ovipositional resource *ad libitum* prior to behavioral testing. For transfer into the behavioral arena, four to six females of a single population were collected in 20 mL glass scintillation vials by a gloved hand and held for no more than one hour. Upon release from their scintillation vials, females were monitored by two observers for 15 min or until all landed and tapped a host with the labellum. After landing on a host, but prior to blood feeding, females were removed from the arena by mouth aspirator. All females that responded to a host on day one of testing were held individually in scintillation vials with access to 10% sucrose and tested again on each of two subsequent days.

### 2.3. Chicken vs. Human Choice Landing Assays: Data Analysis

To analyze our landing assay data, we constructed mixed logistic regression models with binomial error structures using the lme4 package (v. 1.1-14) [61] in R (v. 3.3.2; R Foundation for Statistical Computing, Vienna, Austria). A statistically significant impact of the population on individual mosquito response was determined by model reduction, where models with and without the fixed effect of population were compared by likelihood ratio test (lmtest v. 0.9-35) [62]. Initially, we examined whether the overall response rate to any host varied by mosquito population on day one. A random effect of the “chick” was included in our model to account for inter-individual variation in attractiveness among avian hosts used in the assay [63,64]. Our full model examined the response by the *i*th mosquito to the investigator’s hand and/or the *j*th chick:

Pr[y*_i_* = 1] = logit^−1^(*β*_0*ij*_ + *β*_1_Population_*ij*_ + *u*_0*ij*_) (1)

for 1 ≤ *i* ≤ *n* and 1 ≤ *j* ≤ *m*

where *u*_0_ ∼ N(0, *σ*^2^_j_) represents the random effect of the chick.

This analysis was performed three times: (1) for all populations examined in our behavioral assay, (2) for populations from AG collections only, and (3) for populations from BG collections only.

We then examined whether human host acceptance varied by mosquito population over multiple days of testing. For this, landing events on the human and chick were scored as 1 and 0, respectively. Two fixed effects were considered in the full model: (1) the population from which the mosquito originated, and (2) the day, corresponding to days one through three of testing, which accounted for repeated measurement of individuals. Our full model examined the response by the *i*th mosquito to the investigator’s hand and/or the *j*th chick:

Pr[y*_i_* = 1] = logit^−1^(*β*_0*ij*_ + *β*_1_Population*_ij_*+ *β*_2_Day*_ij_* + *u*_0*ij*_) (2)

for 1 ≤ *i* ≤ *n* and 1 ≤ *j* ≤ *m*

where *u*_0_ ∼ N(0, *σ*^2^_j_) represents the random effect of the chick.

This model was also run 3 times, as described above—once for all populations, once for AG, and once for BG populations—to quantify within and between collection site variation.

Finally, we examined whether the probability of host-switching for an individual female varied according to population. Individuals that switched hosts at least once per multi-day testing period were scored as a 1, and individuals who did not switch hosts but responded at least twice were given a score of 0. Individuals that failed to respond on multiple days were not included in this analysis. Our full model examined the effect of the population on host switching by the *i*th mosquito, as follows:

Pr[y*_i_* = 1] = logit^−1^(*β*_0*i*_ + *β*_1_Population*_i_*+ *u*_0*i*_) (3)

for 1 ≤ *i* ≤ *n*

where *u*_0_ ∼ N(0, *σ*^2^).

For this model comparison, we did not include a random effect of the chick, as for previous models. Many fewer mosquitoes responded during multiple days of testing, which reduced the number of observations per chick.

For all responses, which included overall response rates, host choice, and host-switching, population means were calculated and presented alongside 95% non-parametric bootstrapped confidence intervals (CIs; n boots = 5000).

### 2.4. RNA Sequencing of Mosquito Heads

As a first step toward elucidating the molecular basis for differences in host-seeking behavior, we compared gene expression in the heads of avian-seeking AG2 females with human-seeking BG1 females. These two populations showed statistically significant differences in host acceptance behavior according to their non-overlapping 95% CIs (Table S2). Using these populations, we generated three treatment groups: nulliparous AG2, parous BG1 females, which had deposited their first egg raft, and gravid BG1 females, who had yet to deposit their first raft. Mosquito rearing details for our RNA-seq experiment are included in the Supplemental Methods. Seven- to nine-day-old females were sacrificed at −80 °C between two to six hours after the onset of scotophase, the time of day they are most likely to engage in host-seeking behavior [65]. Four replicate groups of 30 heads bearing intact chemosensory appendages (i.e., antennae, maxillary palps, and the labellum) were dissected on a small petri dish filled with dry ice for each treatment. Heads were held in sterile 1.5 mL microcentrifuge tubes containing Trizol and stored at −80 °C until isolation of RNA by Zymo Direct-zol RNA MiniPrep (Zymo Research, Irvine, CA, USA). RNA quality values (RQI) were assessed by an Agilent 2100 Bioanalyzer (Agilent Technologies Inc., Santa Clara, CA, USA) prior to RNA-seq library preparation and were >6.8 for all pools (Table S3). Complementary DNA synthesis and Illumina Truseq Nano LT library preparation were conducted at the North Carolina State University Genomic Sciences Laboratory according to standard protocols, and indexed libraries were sequenced as 150 bp paired-end reads on a single Illumina NextSeq500 flow cell (Table S3).

### 2.5. Read-Filtering and Quality Control

We assessed read quality using FastQC v.0.11.5 [66] and then removed adapters as well as filter-trimmed reads based on Phred quality scores (Trimmomatic v.0.38) [67]. A sliding window approach was used for filter-trimming, where window size was set to 5 base pairs (bp), the Phred quality threshold was 20, and only properly paired reads at least 50 bp in length were retained. Filtered-trimmed reads were aligned to the *Culex quinquefasciatus* reference genome (VectorBase version 2; 21 August 2019) using STAR v.2.7.6 [68] with ENCODE standard options. We counted aligned reads that met our threshold for mapping quality of 20 or higher using HTSeq-Count [69]. For our downstream analyses, we only considered genes with at least 10 reads mapped in at least 4 samples.

A principal component analysis (PCA) was used to examine sample-to-sample variation in gene expression profile, with the expectation that BG1 samples in different physiological states (gravid vs. parous) should be more similar to one another than BG1 samples were to the AG2 samples. Bayesian generalized linear models were developed with the arm package (v. 1.11-2) [70] to examine whether the top three principal components (PCs) could predict the population (BG1 vs. AG2) and physiological state (gravid vs. host-seeking) of our samples. PC prediction of population or physiological state was determined by simulating a posterior distribution for each PC coefficient in our models (n = 10,000 simulations) and calculating the 2.5% and 97.5% quantiles (i.e., 95% credible intervals). Overlap of these credible intervals with zero indicated that PC could not predict a sample’s population or physiological state. Spearman’s rank correlation coefficients were calculated to examine pairs of samples for gene expression profile correlation and confirm results from our PCA.

### 2.6. Differential Gene Expression Analysis

We quantified differential gene expression (DGE) for each pairwise treatment comparison using DESeq2 [71]. A standard adaptive shrinkage estimator from the ashr package [72] was applied to our log_2_ fold change (FC) values to remove noise due to genes with low read counts. A gene was considered to be differentially expressed if the Benjamini and Hochberg adjusted *p*-value was <0.05 and the absolute shrunken log_2_ FC was 0.58 (1.5 -FC) or greater. To determine whether these differentially expressed genes were associated with specific biological processes, we conducted an overrepresentation analysis with PANTHER (v. 15.0) [73]. Using GO-slim Biological Process annotations, we compared whether specific gene ontology categories were differentially expressed more often than expected for our overall gene set using a Fisher’s exact test with a Benjamini-Hochberg correction for false discovery rate.

### 2.7. Splice Site Variation

To examine splice site variants for BG1 and AG2 populations, we used the splice junction output tables generated by STAR for each sample. We considered high-quality splice junctions to be those (1) present in all samples from a single population using (2) stranded read mappings at (3) annotated genes with (4) at least 100 mapped reads in all populations. We conducted pairwise comparisons between BG1 parous and BG1 gravid females as well as BG1 parous and AG2 females to identify high-quality putatively population-specific splice junctions as compared to the total splice junctions detected (i.e., present in any sample) in the alternate population. Population-specific splice junctions were compared with DGE results for each pairwise comparison to identify whether changes in expression were correlated with splice site variation. A Chi-squared test was used to determine whether the numbers of genes with population-specific splice junctions differed significantly for each pairwise comparison (α = 0.05). These tests assumed that there were 7572 total genes in the BG1 gravid comparison to BG1 parous and 7480 total genes in the BG1 parous comparison to AG2 that met our filtering criteria above.

### 2.8. Candidate Sensory Gene Identification

For our analysis of sensory genes, we focused on the contrast between human-preferring BG1 parous females and avian-preferring AG2 females because both treatments were in a physiological state compatible with host-seeking. Genes from families with known roles in chemosensation and perception of visual cues, and which could explain differences in host preference for these populations, were specifically examined for evidence of DGE. We identified the total numbers of detectable genes from the olfactory receptor (OR), ionotropic receptor (IR), gustatory receptor (GR), odorant binding protein (OBP), sensory neuron membrane protein (SNMP), and chemosensory protein (CSP) families as well as opsins involved in vision. This included any gene from these families with statistically significant differences in expression, regardless of FC. To identify whether these candidate genes were evolutionarily conserved among mosquito taxa, we identified their orthology group (OG) using the OrthoMCL database (v. 6.2) [74]. Genes considered to have significant protein sequence homology had alignment E-value cutoff scores of less than 1.0 × 10^−25^. A subset of these genes with statistically significant differences in expression was validated by quantitative polymerase chain reaction (qPCR; see Supplemental Methods).

## 3. Results

### 3.1. Host Response Rates and Preferences of Cx. pipiens

#### 3.1.1. Population-Level Day One Overall Response Rates

**Figure 1:**
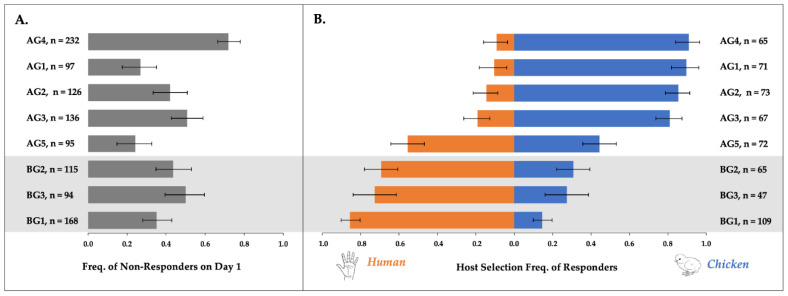
Observed response rates with bootstrapped 95% confidence intervals (n = 5000) to human vs. chicken host for five above-ground and three below-ground populations of *Culex pipiens*. Panel (**A**) shows the frequency of non-responders on Day 1, and n = total number of mosquitoes assessed (d.f. = 7; χ2 = 26.8; *p* < 0.0004). Panel (**B**) shows the frequency with which Day 1 responders (n) selected either a human or avian host in multi-day tests (d.f. = 7; χ2 = 143.46; *p* < 0.0001).

In total, the host acceptance behaviors of 686 AG and 377 BG females were assessed, and of these, 348 AG (50.7%) and 221 BG (58.6%) females accepted a host on day one. For one BG population, BG1, we examined both overall response and host acceptance rates for females at two and three weeks PE and found no statistically significant difference between them (Table S4). Therefore, all BG1 data, regardless of PE time, were pooled for subsequent behavioral analyses. Mean overall response rates on day one varied significantly according to population (d.f. = 7, χ2 = 26.8, *p* < 0.0004; Figure 1A, Table S2). The percentages of BG1, BG2, and BG3 females that responded to any host on day one were 64.9%, 56.5%, and 50.0%, respectively. These overall response rates did not vary significantly among BG populations (d.f. = 2, χ2 = 1.9, *p* = 0.3962). Yet among AG populations, the percentages of females that accepted a host on day one varied significantly (d.f. = 4, χ2 = 29.5, *p* < 0.0001), ranging from 28.0–75.8% (Table S2).

#### 3.1.2. Multi-Day Host Responses by Individual Females

Multi-day responses to the human and avian hosts were quantified for all females that landed on any host on the first day of testing. Human host acceptance varied significantly among AG and BG populations (d.f. = 7, χ2 = 143.46, *p* < 0.0001; Figure 1B). All three BG populations were biased toward human landing, but the strength of the bias varied (d.f. = 2, χ2 = 8.9226, *p* = 0.0116): the percentages of human responses were 69.2%, 72.6%, and 85.4%, for BG2, BG3, and BG1, respectively. Four of the five AG populations were biased toward avian landing, with >80% of females selecting the chicken. Avian response rates were 81.0%, 85.5%, 89.6%, and 90.9% for AG populations AG3, AG2, AG1, and AG4, respectively. The strength of the bias did not differ among these four AG populations, based upon their overlapping 95% CIs. Females belonging to AG5, a population from New York, showed no clear patterns of host acceptance, however, and they landed on the chicken only 44.4% of the time. This population contributed to a statistically significant variation in host landing rates among AG populations (d.f. = 4, χ2 = 36.803, *p* < 0.0001).

#### 3.1.3. Multi-Day Host Switching by Individual Females

The percentages of females that alternated hosts at least once during the multi-day assessment differed significantly by population (d.f. = 7, χ2 = 32.087, *p* < 0.0001). In the three human-seeking BG populations, the percentages of females landing on both the chicken and human host over the three day testing period were 21.6%, 30.0%, and 45.2% for BG1, BG3, and BG2, respectively. In the four avian-seeking AG populations (AG1–AG4), a lower percentage of females alternated hosts: 24.4%, 5.6%, 12.5%, and 0.0%, respectively. Females from NY (AG5), which previously showed indiscriminate host landing behaviors (Figure 1B), had the highest percentage of females to switch hosts across test days, at 51.4%.

### 3.2. Read Quality and Sample Clustering

**Figure 2:**
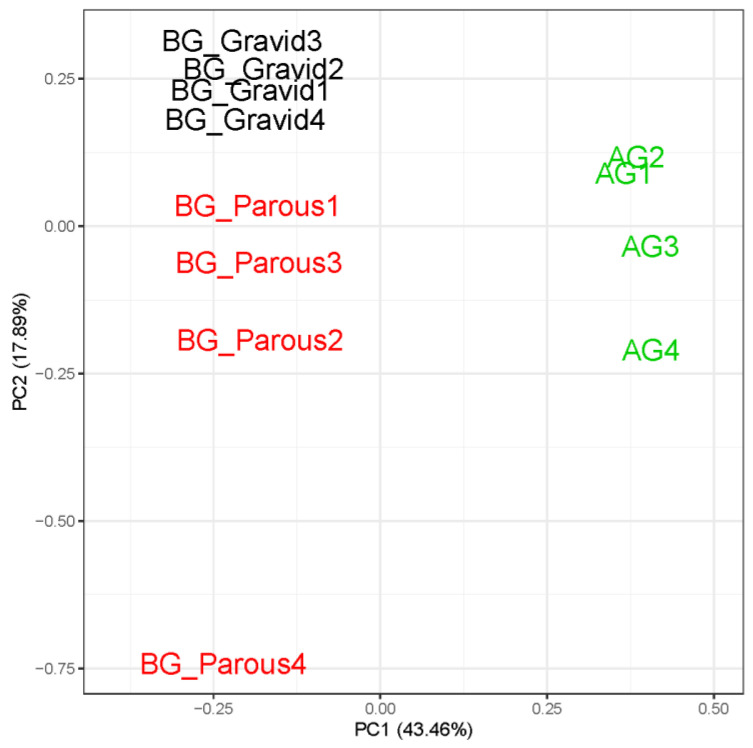
Principal component analysis (PCA) of sample-to-sample variation in gene expression profile at 12,710 genes for below-ground females (BG1) that are parous and gravid as well as above-ground females (AG2) that are nulliparous.

Sequencing of avian-seeking AG2 females and human-seeking BG1 females produced a total of 360,448,777 PE Illumina raw reads, and of these, 314,987,901 (87.4%) remained after filter-trimming. The filtered average per sample read count was 26,248,992 (s.e.m. = 310,695; Table S5). The CpipJ2 official gene set available from VectorBase contained 19,793 genes, and 12,710 (64.2%) could be detected in our dataset based on our filtering criteria of 10 mapped reads per gene in at least four samples. A PCA of the expression profiles at these 12,710 genes confirmed that variation was greater between treatment groups than within (Figure 2). BG1 samples always clustered separately from AG2 samples along PC1, which explained 43.5% of the overall variation in the gene expression profiles for our dataset. Gravid (BG1 gravid) and host-seeking (BG1 parous and AG2) samples clustered separately on PC2, which explained 17.9% of the variation in our dataset. The relationships between PC1 and population as well as between PC2 and physiological state were supported by Bayesian generalized linear model coefficients, whose 95% credible intervals did not overlap with zero (Table S6). Spearman’s Rank correlation coefficients calculated for pairs of samples further supported our PCA and indicated that gene expression profiles were more highly correlated within treatment (Figure S1).

### 3.3. Differential Gene Expression in the Heads of Behaviorally Divergent Females

**Figure 3:**
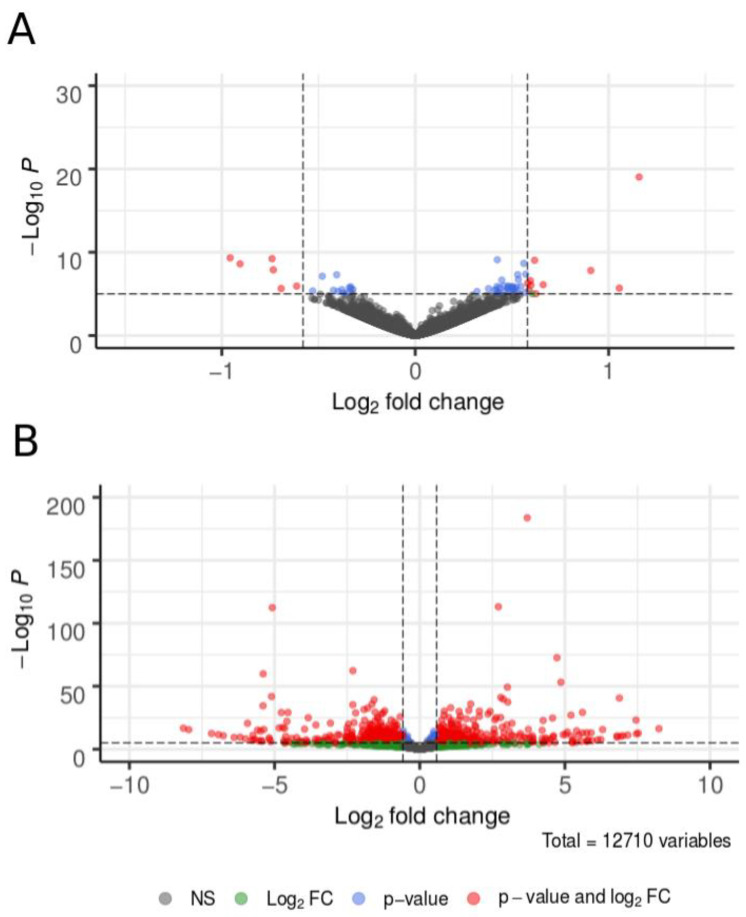
Differential gene expression (DGE) of 12,710 genes in the heads of below-ground *Culex pipiens* (BG1) and above-ground *Cx. pipiens* (AG2). Panel (**A**) shows DGE in a contrast between gravid and parous BG1 females. Panel (**B**) shows the contrast between parous BG1 and nulliparous AG2 females, both ready to engage in host-seeking behavior. Each point represents differential expression of a single gene, where grey points indicate no significant differences in expression, green points show log_2_ fold-change values that are not statistically significant, blue points represent genes with statistically significant differences in expression but low log_2_ fold-change values, and red points represent genes with a >1.5-fold difference between treatments where this difference was statistically significant.

The numbers of statistically significant differentially expressed genes (DEGs) were 544 for the BG1 gravid vs. parous comparison, 2832 for the BG1 parous vs. AG2 nulliparous comparison, and 3011 for the BG1 gravid vs. AG2 nulliparous treatment comparison. When we applied an additional FC threshold (FC > 1.5), the number of DEGs dropped to n = 16, n = 1394, and n = 1402, respectively (Figure 3; see Data S1 for the full list of DEGs). The number of up- and down-regulated DEGs were similar within each treatment contrast, with no strong biases toward up or down regulation in any contrast (Table 2).

Overlap and uniqueness of DGE patterns among the three contrasts indicated which DEGs were population-specific, or specific to physiological state (gravid versus host-seeking). A total of 453 genes were consistently upregulated and 457 genes were downregulated in AG2 females relative to BG1 females, regardless of their physiological state, consistent with population-specific differences in gene expression. Of these, only 253 genes were upregulated and 231 were downregulated in AG2 females relative to BG1 parous females in a physiological state compatible with host-seeking (Figure S2). Three genes were consistently downregulated and two consistently upregulated in BG1 gravid females relative to both AG2 and BG1 parous host-seeking females, suggesting they play an important role in regulating behaviors of mosquitoes in gravid versus host-seeking states (Figure S2).

GO-slim enrichment analysis of the 16 DEGs for the BG1 gravid vs. parous contrast did not identify statistically significant over-representation of any functional gene set. This small number of DEGs included a uricase (CPIJ003456), a xanthine dehydrogenase (CPIJ004365), 4-hydroxyphenylpyruvate dioxygenase (CPIJ004417), a threonine dehydrogenase (CPIJ008256), an allantoicase (CPIJ012990), and an aminomethyltransferase (CPIJ014981), all of which were downregulated in BG1 gravid females. Upregulated genes in BG1 gravid females included E3 ubiquitin-protein ligase highwire (CPIJ003142), *numb* (CPIJ004690), a dual specificity protein phosphatase (CPIJ008018), a camp-specific 3,5-cyclic phosphodiesterase (CPIJ008747), *heat shock protein 83* (CPIJ011244), *cabut* (CPIJ015908), and mitochondrial protein MAS5 (CPIJ018848). The strongest difference in gene expression occurred at *cabut*, whose expression was ca. two-fold higher in gravid BG1 females. Yet DEGs from our contrast of host-seeking BG1 parous to AG2 females were significantly enriched for those involved in sensory perception of chemical stimulus (GO:0007606; padj = 0.0197), sensory perception (GO:0007600; padj = 0.0202), and small molecule catabolic processes (GO:0044282; padj = 0.0479). Because we were interested in identifying genes associated with mosquito responses to vertebrate hosts, further analyses of DEGs focused on families known to be associated with perception of visual cues and chemical volatiles.

### 3.4. Splice Site Variation

On average, STAR detected 5,758,457 (±97,722 s.e.m.), 5,844,577 (±150,889 s.e.m.), and 5,607,207 (±37,538 s.e.m.) total splice junctions for BG1 gravid, BG1 parous, and AG2 females, respectively. A modestly higher and more variable number of reads mapped uniquely for BG1 relative to AG2 females (Table S5), which had the potential to explain the higher mean and standard error for the numbers of splice junctions in BG1 treatments. When we divided the numbers of splice junctions by uniquely mapped reads, however, AG2 had a higher mean number of splice junctions per uniquely mapped read: 0.346 (±0.003 s.e.m.) as compared to 0.336 (±0.004 s.e.m.) and 0.345 (±0.002 s.e.m.) in BG1 parous and gravid treatments, respectively. Filtering STAR-detected junctions for uniqueness by population and treatment as well as on strandedness and average normalized read count (>100 per gene) dramatically reduced the number of population- and treatment-specific splice junctions. We examined two pairwise comparisons: BG1 gravid vs. parous, and BG1 parous vs. AG2. For the first comparison, we identified 28 BG1 gravid-specific splice junctions found in 26 genes, and two BG1 parous-specific splice junctions in two genes. A Chi-square test (χ2 = 20.6, *p* < 0.0001) showed that BG1 gravid females had greater splice site variation than did parous females. For the second comparison, 27 BG1 parous specific splice junctions were found in 26 genes, and 98 AG2 specific splice junctions were found in 94 genes. A Chi-square test (χ2 = 38.8, *p* < 0.0001) indicated that AG2 females had greater splice site variation than did BG1 parous females. A full list of population-specific splice junctions can be found in Data S2. Most of these did not occur at genes with evidence of DGE. For the BG1 gravid vs. parous, no splice variation was observed at any DEG. For the BG1 parous vs. AG2 comparison, however, splice variants were detected at two DEGs in BG1 parous females and at 18 DEGs in AG2 females (Table S7).

### 3.5. Candidate Sensory Gene Analysis

In total, our filtered RNA-seq dataset contained 10 OR, 10 IR, 10 GR, 42 OBP, 2 SNMP, and 11 CSP genes as well as eight opsin genes. Of these, many were differentially expressed according to the adjusted *p*-value, although fewer had 1.5-fold greater or lower expression in AG2 relative to BG1 parous females (Table 3). One OR orthologous to *Cx. quinquefasciatus OR137*, one unnamed GR, and *SNMP1a* were all downregulated in AG2 females. In contrast, all differentially expressed CSPs were upregulated in AG2 females, where the increase in *CSP4* expression was <1.5-fold, increases in *CSP2* and *CSP23* expression were moderate (2.6- and 1.7-fold, respectively), and the increase in *CSP13* expression was highest (26-fold). All of these genes shared significant protein sequence homology with genes from other mosquito species, according to OrthoMCL-db.

Twenty-two of the 42 OBPs detected in our dataset were differentially expressed, and 18 of them had at least a 1.5-fold difference in expression between host-seeking BG1 parous and AG2 females (Table 3). Twelve were downregulated in AG2 females, 10 of which showed at least 1.5-fold lower expression. The remaining 10 OBPs were upregulated in AG2 females, where eight had at least 1.5-fold higher expression. The greatest expression difference occurred at *OBP10*, where expression was detected in all AG2 samples but never detected in BG1 parous females. Seventeen of these 22 differentially expressed *Culex* OBPs shared significant protein sequence homology with OBPs from other mosquito species, while five were found only in *Culex*.

We detected eight opsin genes in our DGE dataset: four long-wave sensitive opsins (*GPROPs 1, 5, 6, 7*), one short wave sensitive opsin (*GPROP2*), one UV detecting opsin (*GPROP3*), a pteropsin ortholog (*GPROP12*), and an ortholog of the conserved Rh7 (*GPROP4*). Statistically significant differences in expression-level were detected at seven of these opsins when we compared AG2 and parous BG1 females. All opsin genes not only shared significant protein sequence homology with those from other mosquito species, but also with insects from other families.

Six sensory genes from Table 3 were selected for qPCR validation of our DGE results (Table S8). Of these, *OBP10*, *CSP2*, *CSP4*, and *GPROP12* were found to be more highly expressed in the avian-seeking AG1 population, while *OBP2* was more highly expressed in the human-seeking BG1 population. These results agreed with fold change trends observed in our DGE analysis, with a single exception, *GPROP1* (Table S9).

## 4. Discussion

We observed significant variation in host responses among North American *Cx. pipiens* collected from above- and below-ground sites, as has been described for other mosquito species [1]. All BG populations showed consistent day one overall response rates (>50.0%) and a higher, albeit significantly variable percentage of landings on the human host (69.2–85.4%). BG1 females had the highest rates of human landing, comparable to results from [23], when they were previously studied. Interestingly, BG2 females, which were derived from the same initial collection [37] but were reared separately and never offered a blood meal, had lower overall host response rates than BG1 and the lowest human landing rates (69.2%) of all three BG populations. While it is possible that the initial split of BG1 from BG2 resulted in an uneven allocation of “human-seeking” alleles to the founders BG1, these differences could also be due to the unique rearing regimes experienced by these populations in the years following their split.

Among AG populations, there was significant variation in both day one overall response rates (range = 28.0–75.8%) and human landing rates (range = 9.1–55.6%). The population from Laurel, MD (AG4), had the lowest day one response rates, while AG5 from New York City, NY, had the highest. AG1-3 populations collected from three different sites in metropolitan Chicago, IL, showed avian acceptance rates > 80%. This agreed with previous blood meal analyses of field-collected AG *Cx. pipiens* from metropolitan Chicago showing that they are predominantly avian-feeding [56,75,76]. AG4 showed the strongest bias toward landing on the chicken (90.9%), yet was not significantly different from AG1–AG3. Less than one quarter of the tested females from AG1–AG4 switched hosts during multi-day testing. Yet AG5 from NY, which showed no strong patterns of landing on either the avian or human host, frequently alternated hosts over multiple days of testing. It is unclear whether behavioral differences observed for AG5 relative to other AG populations were related to differences in their previous rearing regime or natural genetic variation that existed between populations prior to collection. Previous blood meal analyses of *Cx. pipiens* from the Borough of Queens in New York City showed little evidence of mammal feeding [47]. But avian-preferring AG1 and opportunistic AG5 were both established by the CDC in Fort Collins, CO, and reared under similar conditions both there and in our hands. The source of variation in host preference for AG5 relative to other AG populations remains unresolved, but such within-species variation has been observed for other mosquito taxa [1].

Previous work has indicated a genetic basis for mosquito host preference [15,74,75,76,77,78,79], including for *Cx. pipiens* [23]. Therefore, we used RNA sequencing as a first step toward quantifying gene expression differences and splice site variation for one avian- (AG2) and one human-seeking (BG1) population of *Cx. pipiens.* Furthermore, we examined gene expression changes and splice site variation that occur in facultatively autogenous females (BG1) as they transition from a gravid to a parous host-seeking state. Our initial comparison of BG1 gravid versus parous females identified several hundred genes with statistically significant differences in expression, but only 16 with greater than 1.5-FC. Of these 16, five were consistently up- or down-regulated in gravid females relative to host-seeking females of both BG1 and AG2, suggesting that they may regulate blood feeding behaviors or physiological preparedness for blood meal digestion. We also identified splice site variation that appeared to be specific to gravid and parous physiological states in BG1. Twenty-six genes expressed by gravid females contained unique splice junctions not found in parous females. In comparison, only two genes expressed by parous females contained splice junctions not found in gravid females. This raises the intriguing possibility that alternative splicing may play a role in inhibition of blood feeding behaviors while females are gravid. None of the 16 differentially expressed genes described above contained splice junctions unique to gravid or parous females, however.

Differentially expressed genes in the comparison of BG1 gravid and parous females included a uricase (CPIJ003456), 4-hydroxyphenylpyruvate dioxygenase (CPIJ004417), and a threonine dehydrogenase (CPIJ008256), which were downregulated in gravid females, as well as *cabut* (CPIJ015908) and mitochondrial protein MAS5 (CPIJ018848), which were upregulated in gravid females. Free amino acid metabolism and excretion of the nitrogenous waste are critical physiological processes for blood feeding insects. In a close relative, *Cx. pipiens pallens*, free amino acid concentrations are detected in the hemolymph of blood feeding females as quickly as four hours post-blood-meal [80]. Perhaps the expression of 4-hydroxyphenylpyruvate dioxygenase and threonine dehydrogenase, which are critical to phenylalanine/tyrosine [81] and threonine [82] metabolism, respectively, increases in host-seeking females relative to gravid females in preparation for a blood meal and the subsequent amino acid metabolism. Likewise, uricase, which plays a critical role in the degradation of uric acid during and after a blood meal [83], was also higher in host-seeking females. Upregulation of all three genes has even been detected in chemosensory tissues [84,85]. Interestingly, both xanthine dehydrogenase and allantoicase, with metabolic and excretory functions, respectively [83,86], were both more highly expressed in host-seeking BG1 parous females as compared to BG1 gravid females. Yet expression of these genes was lower in AG2 females than in BG1 gravid females. Perhaps there are form-specific interactions with the physiological state that can explain the different expression patterns of these latter two genes.

Upregulation of the transcription factor *cabut* (CPIJ015908) by females in a gravid state was also of interest due to its involvement in diverse physiological and developmental processes. In *Drosophila melanogaster*, *cabut* is involved in growth control, sugar metabolism, and regulation of the circadian clock [87]. It is inducible by 20-hydroxyecdsone (20E) during metamorphosis [88] as well as by sugar-feeding, when both *cabut* and a second transcription factor, *sugarbabe*, are strongly upregulated by Mondo-Mlx [87]. In our *Cx. pipiens*, there was no indication that sugar-feeding was the cause of the differences in the expression of *cabut*; *sugarbabe* (CPIJ007837) expression patterns did not correspond to *cabut* expression patterns among our treatments. Instead, it is known that ovarian ecdysteroidogenic hormone (OEH) production following a blood meal triggers the ovaries of female mosquitoes to produce 20E [89,90]. Furthermore, in the facultatively autogenous mosquito, *Georgecraigius atropalpus*, OEH is expressed after adult emergence and triggers the 20E hormonal cascade without a blood meal [91]. Perhaps the 20E hormonal cascade that occurs PE in our facultatively autogenous BG1 population triggers the expression of *cabut*. Future work should be directed at the consequences of *cabut* expression for blood feeding and the reproductive behaviors of this vector species.

When we compared gene expression profiles for human-seeking BG1 and avian-seeking AG2, both in a physiological state compatible with host-seeking, we identified 1394 genes with statistically significant differences in expression and with a FC of at least 1.5. It is possible that greater FCs and more differentially expressed genes would have been detected had females been used directly from the behavioral assay after landing on human and avian hosts, rather than simply pooling by population. Further studies could confirm this. Along with expression level differences, we also detected splice site variation at dozens of expressed, annotated genes in this pair of populations (Data S2). While gene up- and down-regulation did not appear to strongly differ for human-seeking BG1 and avian-seeking AG2 (Table 2), splice site variants did. AG2 had three times more population-specific splice variants (98 in 94 unique genes) than did BG1 parous females (27 in 26 unique genes). Read counts in AG2 were slightly lower than for BG1 (Table S5), ruling out the possibility that more AG2 variants were detected as a result of deeper sequencing. Instead, there may be greater genetic variation in our AG populations relative to BG1, giving rise to more alternative splice sites. This is consistent with previous studies of genetic variation in these populations [30].

Given the importance of volatile cues to host detection [17], we specifically examined whether any chemosensory genes were differentially expressed in this pair of populations. *Cx. quinquefasciatus* has a chemosensory suite consisting of 180 ORs, 73 IRS, 69 GRs, 109 OBPs, 2 SNMPs, and 27 CSPs, and we expected *Cx. pipiens* to have similar numbers based on their close evolutionary relationship with *Cx. quinquefasciatus*. In our dataset, we were able to detect a portion of these genes: 10 ORs, 10 IRs, 10 GRs, 42 OBPs, 2 SNMPs, and 11 CSPs. This was due to our sequencing strategy. Targeting the whole heads of these behaviorally divergent populations, which bore both the chemosensory appendages and compound eyes, allowed us to quantify differences in expression of genes involved in vision. We identified several opsins that were significantly but modestly upregulated (*GPROP2,3,6,7,12*) in our avian-seeking population, and the differential expression of one of these genes, *GPROP12*, was confirmed by qPCR. For this family, it is possible that differences in lifestyle (above- vs. below-ground) have selected for the observed differences in gene expression, but future work is needed to demonstrate what role, if any, these genes play in behavioral differences between above- and below-ground *Cx. pipiens*.

Sequencing of whole heads to include compound eyes was carried out at the expense of sensitivity for lowly expressed chemosensory genes. Yet even at this coarse resolution, we detected statistically significant differences in expression at dozens of chemosensory genes (Table 3). As one example, *OR137*, which has the potential for involvement in *Cx. quinquefasciatus* host-seeking behaviors due to its antennal expression [92] as well as downregulation following a blood meal [85], was strongly downregulated in our avian-seeking females relative to human-seeking females. An unnamed GR (CPIJ011564) was also significantly downregulated in our avian-seeking AG2 population, but its tissue specificity and expression patterns pre- and post-blood meal are not known. *IR76b*, which is expressed in the antennae of *Cx. quinquefasciatus* [84] and whose homologue in *Anopheles gambiae* responds to butylamine [88], was modestly upregulated in our avian-seeking *Cx. pipiens*. Avian-seeking *Cx. quinquefasciatus* respond to this volatile compound in single sensillum recordings [93], so perhaps higher levels of IR76b expression in our avian-seeking *Cx. pipiens* are part of the mechanism by which they detect their preferred host.

Proportionally, divergence in OBP and CSP expression levels comprised the majority of the chemosensory gene expression differences in our comparison of AG2 and BG1 parous females. Previous studies have shown that genes involved in odorant binding, including OBPs, are among the most rapidly evolving for the above- and below-ground *Cx. pipiens* forms [94]. The roles of these gene families in host detection remain unclear in mosquitoes [17,95], but OBPs are known to influence host preference in phytophagous insect species [96]. This makes the observed differences in expression levels between our human- and avian-seeking populations of particular interest. While the CSP genes detected in our dataset always had homologues in other vector species, several of our OBPs did not (*OBP4*, *OBP10*, *OBP32*, *OBP36*, and *OBP64*). Indeed, some of the strongest expression level differences we saw in the candidate sensory genes were from CSP and OBP gene families, including several OBPs that were *Culex* specific. *OBP10*, *OBP20*, *OBP51*, and *CSP13* had more than four-fold higher expression in our avian-seeking *Cx. pipiens*. *OBP64*, on the other hand, was strongly downregulated in the avian-seeking population. Of these, *OBP10* and *OBP51* are expressed in the antennae of *Cx. quinquefasciatus*, while *OBP20* and *OBP64* can be detected in other tissues [84]. This lack of antennal specificity does not necessarily preclude a role in host detection [96], and future studies should investigate whether changes in expression levels of these genes impact host preference in *Cx. pipiens*.

Three genes relevant to host detection and feeding, but not included in our candidate sensory gene analysis, were also differentially expressed in our human- and avian-seeking *Cx. pipiens*: *acj6* (CPIJ014571), a pickpocket gene (*ppk*; CPIJ007315), and a d7 long form salivary gland protein (CPIJ014550). The transcription factor *acj6* was previously shown to determine the odor specificities of a subset of olfactory receptor neurons in *D. melanogaster* [97], and this gene was moderately but significantly upregulated in the heads of our avian-seeking *Cx. pipiens*. Perhaps the observed differences in expression levels of this transcription factor impact the patterning and expression of receptors on ORNs in our human- and avian-seeking mosquito populations. Work in *D. melanogaster* has also shown that members of the pickpocket family of epithelial sodium channels are involved in the taste system [98], and one unnamed *ppk* was strongly downregulated in our avian-seeking population. Finally, while not necessarily involved in host detection, d7 long form salivary gland proteins are important to mammal feeding in *Cx. quinquefasciatus* [99]. One such protein was significantly upregulated in avian-seeking females, although it was not the homolog of ADP-binding CxD7L1, known to enhance mammal feeding. We speculate that the *ppk* and salivary gland proteins may play a role in the final stages of host acceptance post-landing and perhaps facilitate feeding in our behaviorally divergent populations, although we have not yet studied what, if any, behavioral differences exist between our *Cx. pipiens* populations after landing.

## 5. Conclusions

*Cx. pipiens* is a bridge vector of WNV in North America [75], where avian-seeking behaviors contribute to viral amplification in avian species, and mammal-seeking behavior drives epizootic transmission of the virus into humans. Here we quantified the extent of variation in host preference for *Cx. pipiens* collected from AG and BG breeding and overwintering sites. We identified host landing patterns ranging from highly avian-seeking, which was most typical in AG populations, to human-seeking, which was primarily observed in BG populations. Pairwise comparisons of gene expression from the heads of human-preferring females in gravid and host-seeking states as well as the comparison of human- and avian-seeking females resulted in identification of DEGs with metabolic, chemosensory, vision, and regulatory function. Future functional work will determine the specific roles of these genes in our behaviorally divergent populations.

## Figures and Tables

**Table 1:** Differences in behavioral and physiological traits for the *pipiens* and *molestus* bioforms of the northern house mosquito, *Culex pipiens*.

Trait	Form *Pipiens*	Form *Molestus*	Citations
Overwintering behavior	Diapausing.	Does not diapause.	[31,32,33,34,35]
Breeding site	Breeds in above-ground aquatic habitats.	Breeds in below-ground aquatic habitats.	[36,37,38,39,40]
Mating behavior	Mates in swarms above ground.	Mates below ground in confined spaces.	[22,26,39,41,42]
Reproduction	Requires a blood meal for egg production in the first gonotrophic cycle (anautogeny).	Does not require a blood meal for egg production in the first gonotrophic cycle (facultative autogeny).	[43,44,45,46]
Host preference	Primarily avian-seeking.	Primarily mammal-seeking.	[23,47,48,49,50,51,52,53,54,55,56]

**Table 2:** Numbers of differentially expressed genes that reach our fold change (±1.5) and statistical significance thresholds (α = 0.05) after a Benjamini and Hochberg adjustment for false discovery rate. The reference for each treatment contrast is italicized.

	BG Gravid vs. *BG Parous*	*BG Parous* vs. AG Nulliparous	*BG Gravid* vs. AG Nulliparous
FC > 1.5 (up)	10	705	761
FC < 1.5 (down)	6	689	641
Total	16	1394	1402

**Table 3:** Differentially expressed genes with sensory function in the whole heads of above-ground (AG2) host-seeking females relative to below-ground host-seeking females. *p*-values are adjusted for multiple comparisons using a Benjamini-Hochberg false discovery rate correction. Bolded lines have both a statistically significant Benjamini-Hochberg adjusted *p*-value (padj) and an absolute log_2_ fold change (FC) value greater than 0.58 (equivalent to a 1.5-fold difference).

Gene Family	Vector Base ID	Gene Name	Log_2_ FC (Shrunken)	padj	Orthology Group
Odorant receptor	**CPIJ016433**	**OR137**	**−2.54**	**9.6 × 10^−5^**	**OG6_163622**
Ionotropic receptor	CPIJ019300	IR76b	0.55	3.6 × 10^−3^	OG6_126385
Gustatory receptor	**CPIJ011564**	**NA**	**−1.22**	**2.4 × 10^−2^**	**OG6_187005**
Odorant Binding Proteins	**CPIJ001730**	**OBP4**	**−1.07**	**4.0 × 10^−4^**	**OG6_201790**
	**CPIJ002108**	**OBP108**	**−0.83**	**4.0 × 10^−2^**	**OG6_136613**
	**CPIJ002109**	**OBP107**	**−1.11**	**1.1 × 10^−2^**	**OG6_151055**
	**CPIJ002111**	**OBP110**	**−0.60**	**2.6 × 10^−2^**	**OG6_163322**
	**CPIJ004145**	**OBP64**	**−4.73**	**7.6 × 10^−16^**	**OG6_140589**
	CPIJ004634	OBP102	0.49	6.1 × 10^−3^	OG6_142050
	CPIJ007604	OBP1	−0.49	2.1 × 10^−2^	OG6_117872
	**CPIJ007617**	**OBP2**	**−0.65**	**6.4 × 10^−3^**	**OG6_163124**
	CPIJ008793	OBP6	−0.55	2.0 × 10^−4^	OG6_110106
	**CPIJ009568**	**OBP8**	**0.64**	**1.4 × 10^−4^**	**OG6_110106**
	**CPIJ010367**	**OBP55**	**−1.09**	**1.0 × 10^−12^**	**OG6_124323**
	**CPIJ010787**	**OBP51**	**2.30**	**9.1 × 10^−11^**	**OG6_150797**
	**CPIJ012716**	**OBP17**	**0.89**	**2.1 × 10^−2^**	**OG6_107904**
	**CPIJ012717**	**OBP18**	**1.07**	**2.4 × 10^−2^**	**OG6_150726**
	**CPIJ012719**	**OBP20**	**3.18**	**5.4 × 10^−19^**	**OG6_107904**
Odorant Binding Proteins	**CPIJ013976**	**OBP10**	**8.24**	**6.2 × 10^−15^**	**OG6_153567**
	**CPIJ014525**	**OBP24**	**0.64**	**8.9 × 10^−4^**	**OG6_107904**
	**CPIJ016479**	**OBP32**	**−0.77**	**3.8 × 10^−2^**	**OG6_117689**
	CPIJ016965	OBP28	0.46	5.5 × 10^−3^	OG6_128903
	**CPIJ016966**	**OBP29**	**−1.92**	**2.0 × 10^−29^**	**OG6_128903**
	**CPIJ019610**	**OBP36**	**−0.81**	**1.2 × 10^−2^**	**OG6_167102**
Sensory Neuron Membrane Protein	**CPIJ014330**	**SNMP1a**	**−0.62**	**5.0 × 10^−3^**	**OG6_117830**
Chemosensory Proteins	**CPIJ002605**	**CSP2**	**1.42**	**1.9 × 10^−12^**	**OG6_120812**
	CPIJ002608	CSP4	0.32	4.9 × 10^−2^	OG6_107706
	**CPIJ002618**	**CSP13**	**4.66**	**6.3 × 10^−5^**	**OG6_120813**
	**CPIJ002628**	**CSP23**	**0.78**	**7.3 × 10^−6^**	**OG6_162766**
Opsins	**CPIJ004067**	**GPROP1**	**−0.60**	**1.3 × 10^−3^**	**OG6_163080**
	CPIJ005000	GPROP2	0.24	3.1 × 10^−2^	OG6_120825
	CPIJ009246	GPROP3	0.19	4.5 × 10^−2^	OG6_124296
	CPIJ011571	GPROP6	0.50	9.2 × 10^−8^	OG6_104608
	CPIJ011573	GPROP7	0.54	4.7 × 10^−3^	OG6_104608
	CPIJ014334	GPROP12	0.48	3.0 × 10^−2^	OG6_105417

## Data Availability

RNA-seq data have been archived at NCBI BioProject accession PRJNA702543 (https://www.ncbi.nlm.nih.gov/sra/PRJNA702543, accessed on 15 March 2021). Behavioral data and all scripts used for data analysis are available at https://github.com/mcadamme/Culex_RNAseq_Chemosensory/ (accessed on 15 March 2021).

## Supplementary Materials

The following are available online at https://www.mdpi.com/2075-4450/12/3/271/s1, Supplemental Methods describe the analysis of BG1 at two and three weeks PE, mosquito rearing methods prior to RNA sequencing, and qPCR validation of sensory genes. Table S1 shows collection information for each of the eight tested *Cx. pipiens* populations. Table S2 provides means and 95% CIs for overall response rates, human acceptance rates, and rates of host switching for all eight *Cx. pipiens* populations. Table S3 shows RNA quality indicators and Illumina indices used for sequencing. Table S4 shows the results of BG1 behavioral analyses at different PE times. Table S5 contains filtering and alignment statistics for our RNA-seq experiment. Table S6 contains the analysis of PCs 1 and 2 with respect to mosquito population and physiological state. Table S7 shows DEGs with population-specific splice variants. Table S8 contains qPCR primer sequences used for sensory gene expression validation. Table S9 provides qPCR validation results for six selected genes from Table S3. Figure S1 contains Spearman’s rank correlation coefficients for paired sample gene expression profiles. Figure S2 contains a Venn diagram of the numbers of differentially expressed genes for each pairwise comparison. Data S1 contains all differentially expressed genes according to adjusted *p*-value for all pairwise RNA-seq treatment comparisons. Data S2 contains splice junctions unique to each sequenced population.
