# Supplementary material for: Differential Gene Expression in the Heads of Behaviorally Divergent Culex pipiens Mosquitoes
Source: Insects. 2021 Mar 23;12(3):271. doi: 10.3390/insects12030271 (PMC8005152; doi:10.3390/insects12030271)
Supplement: Supplementary file 1 [file insects-12-00271-s001.zip › supplementary-xml/supplementary-xml.pdf]

Supporting Materials

Methods

Analysis of BG1 at two- and three-weeks Post-Emergence

We examined whether the behavior of BG1 females differed at two- and three-weeks post-emergence (PE) using a pair of generalized linear models with binomial error structures in R. We first examined whether day 1 responses of the *i*th mosquito varied according to PE time with the *j*th chick, using the following full model:

Full model to examine overall response:

$$\Pr[y_i=1] = \text{logit}^{-1}(\beta_{0ij} + \beta_1 \text{PE}_{ij} + u_{0ij})$$

For $1 \leq i \leq n$ and $1 \leq j \leq m$

where $u_0 \sim N(0, \sigma^2{}_j)$ represents the random effect of chick.

This was compared to a reduced model without the fixed effect of PE time using a likelihood ratio test from the lmtest package (v. 0.9-35).

A second generalized linear model examined the effect of BG1 PE time on host choice of the ith mosquito, as follows:

Full model to examine individual (i) host response:

$$\Pr[y_i=1] = \text{logit}^{-1}(\beta_{0i} + \beta_1 \text{PE}_i + \beta_2 \text{Day}_i + u_{0i})$$

For $1 \leq i \leq n$

where $u_0 \sim N(0, \sigma^2)$

This model was compared to a reduced model without the fixed effect of PE time using a likelihood ratio test, as well. Individuals were only included in this analysis if they responded on multiple days, and we accounted for repeated measures by including day as a fixed effect in our model. Given the small size of our dataset we were unable to include chick as a random effect.

Mosquito Rearing for Differential Expression Analysis

Mosquitoes of the AG2 and BG1 populations were reared at density of ca. 150 larvae per pan, and each pan was fed a combination of 160 mg liver powder and 80 mg yeast. Pupae were transferred to population-specific 32 × 32 × 32 cm BugDorm-1 cages (MegaView Science Education Services Co., Taichung, Taiwan), and adults were always provided a 10% sucrose solution. Adult mosquitoes and larval pans were held in an environmental chamber at 25 +/–1 degrees C and 50% RH under a 16:8 LD photoperiod. Ovipositional resources were provided to AG2 females, as well as half of the BG1 females, which provided us with parous BG1 that were physiologically ready for host-seeking. The other half of the BG1 females were deprived of an ovipositional resource, which meant that the majority of females were gravid at the time of dissection. We examined the ovaries for a subset of AG2 and all of the BG1 females used sequencing by dissecting the abdomens and scoring the Christopher's Stages of the follicles. For AG2 females 36 of 36 mosquitoes examined possessed ovaries in Christopher's Stage IIa. The ovaries of all females in the BG1 'gravid' and 'parous' groups were assessed, and we only collected heads from females with ovarian follicles in Christopher's Stage V and Christopher's Stage IIa, respectively.

qPCR Validation of Select Genes

We used quantitative PCR to verify the expression patterns of six genes found to be differentially expressed between the behaviorally-divergent populations, AG2 and BG1 (Table 3): CPIJ007617 (OBP2), CPIJ013976 (OBP10), CPIJ002605 (CSP2), CPIJ002608 (CSP4), CPIJ004067 (GROP1), and CPIJ014334 (GROP12).

RNA isolation and cDNA synthesis: Seven to nine day old nulliparous AG2 females and parous BG1 females were euthanized at -80°C, four hours (+/– 15 min) after the onset of scotophase. Samples for each treatment were generated by pooling ten, fully-intact female heads. Total RNA was extracted using TRIzol Reagent (Invitrogen, Carlsbad, CA) according to the manufacturer's instructions with the following additions: phaselock tubes (Quantabio, Beverly, MA) was used during chloroform-phenol separation steps, and 2 uL of GlycoBlue was added during RNA precipitation (Thermo Fisher Scientific, Waltham, MA) to increase pellet visibility. Final RNA yield was assessed using a Nanodrop Lite spectrophotometer (Thermo Fisher

Scientific, Waltham, MA). First-strand cDNAs were synthesized from an input of 250 ng RNA using iScript cDNA Synthesis Kit (BioRad, Hercules, CA) and a blend of oligo(dT) and random hexamer primers. Thermocycler conditions for cDNA synthesis followed manufacturer's instructions (5 min at 25 °C, 20 min at 46 °C, 1 min at 95 °C).

qPCR Primer Design: In the absence of a published genome for *Cx. pipiens*, cDNA sequences used to design primers were obtained from the published genome assembly of the close relative, *Cx. quinquefasciatus*, available from VectorBase. qPCR primers were created using the Integrated DNA Technologies PrimerQuest Tool and designed so that primers/amplicons span at least one exon-exon junction (to prevent amplification of genomic DNA). Additionally, all primers have an annealing temperature of 59 °C to prevent non-specific amplification. The VectorBase Basic Local Alignment Search Tool (BLAST) was used to ensure all primer sequences (Table S8) were specific to their target gene. Additionally, amplicon sizes for each primer set were examined using non-quantitative RT-PCR and gel electrophoresis. Amplicons were run on a 2% agarose gel at 90V for 60 min alongside a GeneRuler 1 kb Plus DNA Ladder (Thermo Fisher Scientific, Waltham, MA) to verify correct base pair length.

qPCR: qPCR reactions were carried out in a Roche LightCycler 480 Real-Time qPCR machine using the Luna Universal qPCR Master Mix (New England BioLabs, Ipswich, MA) in a final volume of 20 mL. qPCR reactions were carried out using 40 cycles of the following thermocycler conditions: 95 °C for 30 s, 56 °C for 15 s, 60 °C for 30 s. Additionally, all samples were technically replicated three times.

qPCR Data Analysis: Fold change in gene expression between populations was analyzed using the ΔΔCt method (Livak et al. 2001). Technical replicate Ct values were averaged to generate mean Cts for calculation of ΔΔCt and fold change. Sensory gene expression was first normalized using 60s ribosomal protein L8 or RPL8 (VectorBase ID: CPIJ000162). RPL8 was selected as the control gene for this study because previous work in our lab consistently found its expression to be uniform across the behaviorally-divergent population. We then determined if the expression differences between AG2 and BG1 populations agreed with the trends for the same genes reported in Table 3.

Supplementary Tables

Table S1. - Collection information for eight study populations of above- and below-ground collected *Cx. pipiens,* including year of population initiation, location of collection, and the size of G0. AG refers to populations collected from above-ground breeding and/or overwintering sites, while BG refers to those populations collected from below-ground breeding sites. BG1 and BG2 were originally collected by Mutebi et al. 2009 but were split and reared under different conditions prior to behavioral analysis. BG1 was reared at Michigan State University and historically offered a blood meal (Fritz et al. 2015), while BG2 was reared at the Centers for Disease Control in Fort Collins, CO, without blood feeding.

**Population ID**	**Collection Year**	**Collection Site**<br>**GPS Coordinates**	**Size of G0**
BG1	2009	41.6502247, -87.600140<br>Calumet Water Reclamation Plant; Chicago, IL	7000 adults and larvae
BG2	2009	41.6502247, -87.600140<br>Calumet Water Reclamation Plant; Chicago, IL	7000 adults and larvae
BG3	2013	37.904445, -122.653184<br>Stinson Beach, California	20 females (wild caught then blood-fed in lab)
AG1	2010	*GPS coordinates unknown*<br>Oak Lawn, IL	200 diapausing adults
AG2	2016	42.029246, -87.70564<br>Evanston, IL	28 egg rafts
AG3	2016	42.094783, -87.770168<br>Northfield, IL	31 egg rafts
AG4	2017	39.111413, -76.903376<br>Laurel, MD	80 egg rafts
AG5	2008	40.65874, -73.9862; 40.7900, -73.7808<br>New York City, NY	300 diapausing adults

Table S2. - By population number of females tested, numbers of responders, percentages of individuals responding to a human vs. chicken host in multi-day, host-choice assays.

Population	Groups of 4-6 females, *n reps*	Total tested, *n*	Total respond-ers, *n*	Day 1 responders, % (2.5, 97.5 quantiles)	Human Acceptance Rate, % (2.5, 97.5 quantiles)	Host Alternating Rate, % (2.5, 97.5 quantiles)
AG1	19	97	71	73.2 (63.9, 81.4)	10.4 (3.9, 18.1)	24.4 (13.3, 37.8)
AG2	24	126	73	57.9 (49.2, 66.7)	14.5 (8.6, 21.4)	12.5 (3.1, 25.0)
AG3	27	136	67	49.3 (41.2, 57.4)	19.0 (12.7, 26.2)	5.6 (0.0, 16.7)
AG4	46	232	65	28.0 (22.4, 33.6)	9.1 (3.4, 15.9)	0.0 (0.0, 0.0)
AG5	23	95	72	75.8 (66.3, 84.2)	55.6 (46.8, 64.3)	51.4 (35.1, 67.6)
BG1	33	168	109	64.9 (57.7, 72.0)	85.4 (80.2, 90.1)	21.7 (11.7, 33.3)
BG2	26	115	65	56.5 (47.0, 65.2)	69.2 (60.6, 77.9)	45.2 (29.0, 61.3)
BG3	19	94	47	50.0 (40.4, 59.6)	72.6 (61.3, 83.9)	30.0 (0.0, 60.0)

Table 3. - RNA quality indicator numbers (RQI) and Illumina indices corresponding to each pool for our differential expression analysis.

Population	Condition	Sample Name	RQI Values	Illumina Index #	Index Sequence
BG1	gravid	M1.1	8.8	1	ATCACG
BG1	gravid	M1.2	8.7	2	CGATGT
BG1	gravid	M1.3	7.8	3	TTAGGC
BG1	gravid	M1.4	7.5	4	TGACCA
BG1	parous	M2.1	7.9	5	ACAGTG
BG1	parous	M2.2	7.2	6	GCCAAT
BG1	parous	M2.3	7.5	7	CAGATC
BG1	parous	M2.4	7.2	8	ACTTGA
AG2	nulliparous	M4.1	6.8	13	AGTCAA
AG2	nulliparous	M4.2	7.8	14	AGTTCC
AG2	nulliparous	M4.3	8	15	ATGTCA
AG2	nulliparous	M4.4	9.1	16	CCGTCC

Table S4. - Tests of statistical significance for BG1 female behavior according to post-emergence (PE) time. The models were described in the Supplementary Methods, and examined probability of overall response (Overall Resp) and probability of human landing (Human Pref) for BG1 females at 2 and 3 weeks PE. Outputs provided below are from the likelihood ratio tests, which compared models with and without PE time as a fixed effect. Lack of statistical significance ($p < 0.05$) indicated PE time did not significantly impact BG1 female behavior.

Behavior	df	Chisq	*p*-val
Overall Resp	1	1.59	0.21
Human Pref	1	0	1

Table S5. - Filtering and alignment statistics for RNA sequencing data.

Sample	Total PE Reads	Filtered PE Reads	% Kept	Uniquely Mapped Reads (UMR)	% UMR	% Total Mapped Reads
M1.1	28500393	24958855	87.6	16082897	64.4	75.9
M1.2	28489985	24938189	87.5	16257039	65.2	76.1
M1.3	30205912	25826507	85.5	16831140	65.2	76.4
M1.4	30638695	26977182	88.1	17536946	65.0	76.5
M2.1	30804792	26868595	87.2	17415613	64.8	75.4
M2.2	29632161	25904361	87.4	16579296	64.0	75.6
M2.3	32484696	28228436	86.9	18224845	64.6	75.4
M2.4	32016817	27950272	87.3	17259453	61.7	76.5
M4.1	29874076	26126664	87.5	16216199	62.1	74.2
M4.2	28842848	25346952	87.9	16065200	63.4	76.1
M4.3	29043561	25540717	88.0	15976815	62.6	75.0
M4.4	29914841	26321171	88.0	16579500	63.0	75.8

Table S6 - Coefficient estimates for principal components (PCs) 1 through 3 generated from six Bayesian generalized linear models, and their respective 95% credible intervals generated by simulating the PC coefficient's posterior distribution (nsims = 10,000). For model responses, strain refers to whether the sample came from BG1 or AG2, and physiological state indicates whether the samples were gravid or host-seeking at the time of RNA collection. Bolded models have PC coefficients with 95% credible intervals that do not overlap zero.

Model	Model Response	Model Predictor	PC Coef Estimate	95% Credible Interval (2.5, 97.5% quantiles)
**1**	**Strain**	**PC1**	**–0.2513**	**(–0.4530, –0.0533)**
2	Strain	PC2	0.0043	(–0.1261, 0.1368)
3	Strain	PC3	–0.0143	(–0.2343, 0.2019)
4	Physiological State	PC1	0.0906	(–0.0332, 0.2138)
**5**	**Physiological State**	**PC2**	**–0.7118**	**(–1.3790, –0.0629)**
6	Physiological State	PC3	–0.1197	(–0.3636, 0.12876)

Table S7. – Differentially expressed genes (DEGs) with population-specific splice variants.

Population with splice variant	Higher Expression	VectorBase Gene IDs
BG1	BG1	CPIJ001456
BG1	AG2	CPIJ000118
AG2	BG1	CPIJ005968
AG2	AG2	CPIJ000699, CPIJ000830, CPIJ002498, CPIJ002549, CPIJ003738, CPIJ004652, CPIJ007749, CPIJ008495, CPIJ011975, CPIJ013437, CPIJ013541, CPIJ013621, CPIJ013920, CPIJ014384, CPIJ014777, CPIJ016929, CPIJ016979

Table S8. - qPCR primers for sensory gene expression validation.

VectorBase ID (Gene Name)	Primer_ID	Primer Sequence 5' - 3'	Amplicon Length (bp)
**CPIJ007617 (OBP2)**	OBP2_F	ACCGAGGCGAGATGCTGAATACC	115
	OBP2_R	GAAGATGCCATCAAGCGCTTCAGC	
**CPIJ013976 (OBP10)**	OBP10_F	CTTGAGTTGATGCAAGGAATG	116
	OBP10_R	GTATCGAAGCCCTCTTAGTAG	
**CPIJ002605 (CSP2)**	CSP2_F	CCAGTACACCGACAAGTTC	77
	CSP2_R	GGATGTAGTTGCTGAGGATG	
**CPIJ002608 (CSP4)**	CSP4_F	CGGAAGGCAAGGAACTAAA	112
	CSP4_R	TCAACGATAAAGTGGATCACC	
**CPIJ004067 (GROP1)**	GROP1_F	CGCCTTTACTGGATCCTTATT	99
	GROP1_R	TTACCGGCCAAACCTTTC	
**CPIJ014334 (GROP12)**	GROP12_F	CTGCTCATCTGGTGTTACTC	118
	GROP12_R	TCAGGGTTTGCGTTTCC	
**CPIJ000162 (RPL8)**	RPL8_F	GCCCTGATTGAACAATGG	145
	RPL8_R	ACTCCCTTCAGGTATCCG	

Table S9. - qPCR validation of a subset of DEGs in the whole heads of above-ground (AG2) host-seeking females relative to below-ground (BG1) host-seeking females. Fold change was calculated using the $2^{-\Delta\Delta Ct}$ method. Expression of OBP10 and GROP12 was not detected* in the heads of BG1 females but expression was detected in the heads of AG2 females.

VectorBase ID (Gene Name)	ΔΔCt	Fold Change ($2^{-\Delta\Delta Ct}$)	Interpretation of qPCR expression differences	Does the direction of FC in expression match across techniques?
**CPIJ007617 (OBP2)**	0.91	0.53	More highly expressed in BG1	Yes
**CPIJ013976 (OBP10)**	NA*	NA*	Expressed in AG2, not detected in BG1	Yes
**CPIJ002605 (CSP2)**	-1.90	3.72	More highly expressed in AG2	Yes
**CPIJ002608 (CSP4)**	-7.47	177.29	More highly expressed in AG2	Yes
**CPIJ004067 (GROP1)**	-0.73	1.66	More highly expressed in AG2	No
**CPIJ014334 (GROP12)**	NA *	NA *	Expressed in AG2, not detected in BG1	Yes

Supplementary Figures

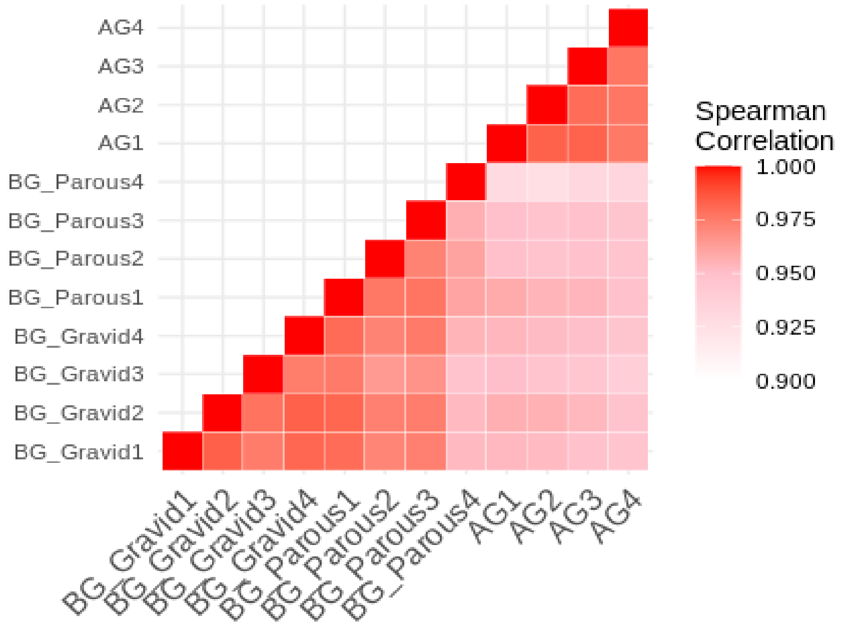

Figure S1. - Spearman's rank correlation coefficients calculated for each pair of samples using the matrix of normalized read counts.

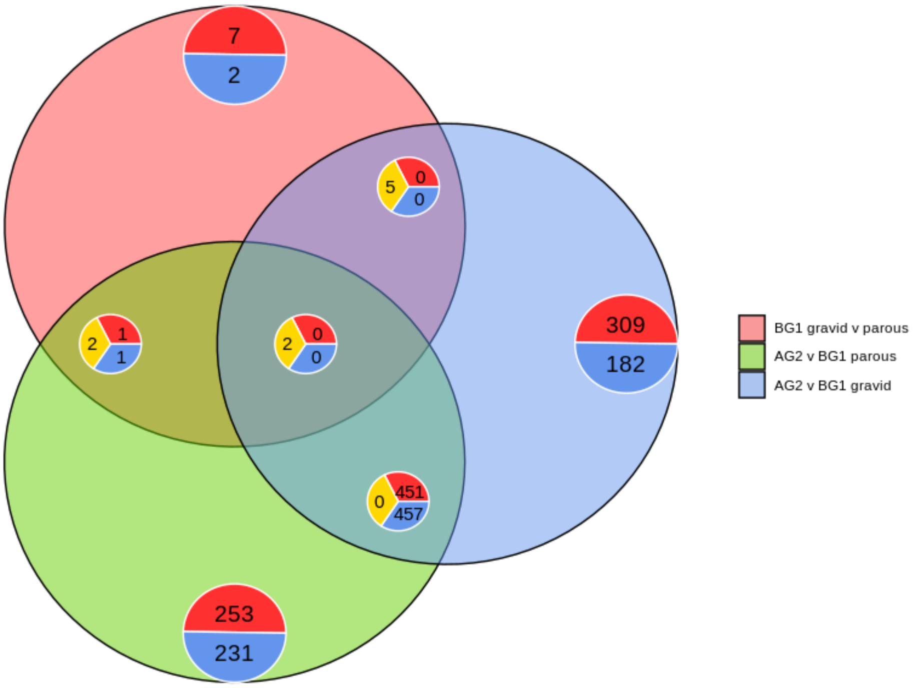

Figure S2. - Patterns of differential gene expression shared between treatment contrasts. Inset pie charts show the numbers of up-regulated (red), down-regulated (blue), or contra-regulated (yellow) genes within each treatment contrast or shared by more than one treatment contrast. Reference levels for fold-change in expression are BG1 parous for contrasts 1 and 2 (red and green circles, respectively), and BG1 gravid (blue circle).

Supplemental References:

1. Livak, K., Schmittgen, T. Analysis of relative gene expression data using realtime quantitative PCR and the $2^{-\Delta\Delta Ct}$ method. *Methods.* **2001**, *25(4)*, 402–408. https://doi.org/10.1006/meth.2001.1262
2. Mutebi, J.P.; Savage, H.M. Discovery of *Culex pipiens pipiens* form *molestus* in Chicago. *J. Am. Mosq. Control Assoc.* **2009**, *25*, 500–503.
3. Fritz, M.L.; Walker, E.D.; Miller, J.R.; Severson, D.W.; Dworkin, I. Divergent host preferences of above-and below-ground *Culex pipiens* mosquitoes and their hybrid offspring. *Med. Vet. Entomol.* **2015**, *29*, 115–123.
